# Regret concerning treatment decisions in patients with primary or secondary brain tumors – a cross-sectional exploratory bicentric analysis

**DOI:** 10.1007/s11060-026-05534-2

**Published:** 2026-03-25

**Authors:** Julia Reuter, Tim Werfel, Alexander Rühle, Georg Wurschi, Anja Mehnert-Theuerkauf, Johannes Wach, Klaus Pietschmann, Tomas Kazda, Maximilian Römer, Nils H. Nicolay, Andreas Hinz, Clemens Seidel

**Affiliations:** 1 Department of Radiation Oncology, University Medical Center Leipzig, Leipzig, Germany; 2 Comprehensive Cancer Center Central Germany, Partner Site Leipzig, Leipzig, Germany; 3 Department of Radiotherapy and Radiation Oncology, Jena University Hospital, Jena, Germany; 4 Comprehensive Cancer Center Central Germany, Partner Site Jena, Jena, Germany; 5 Department of Medical Psychology and Medical Sociology, University Medical Center Leipzig, Leipzig, Germany; 6 Department of Neurosurgery, University Medical Center Leipzig, Leipzig, Germany; 7 Department of Radiation Oncology, Masaryk Memorial Cancer Institute, Brno, Czech Republic

**Keywords:** Malignant glioma, Brain metastasis, Decision regret, HRQoL, Medical care

## Abstract

**Purpose:**

To determine the extent of decisional regret (DR) and contributing factors after treatment of primary and secondary brain tumors (BT).

**Methods:**

Patients who received radiotherapy (RT) for a BT between 2010 and 2024 were eligible for this bicentric cross-sectional study. DR was assessed using the 5-item Decision Regret Scale (DRS). Overall and treatment-specific DR was determined. Besides DR, health related quality of life (HRQoL), distress, anxiety, depression, and satisfaction with medical care were determined. Associations between DR and covariates were examined in univariate analyses. Linear regression models were calculated for multivariable analyses.

**Results:**

From 310 eligible contacted patients, 162 were included. Ninety-four patients (58%) suffered from malignant glioma, and 68 (42%) had brain metastases (BM). Median age was 58 years, median interval between the last RT fraction and study participation 22 months. Thirty-two patients (20%) reported no, 85 (53%) mild, and 45 (28%) strong overall DR. Regarding their decision towards specific treatments, 37 patients (23%) expressed strong DR concerning RT, 45 (30%) regarding chemotherapy, and 29 patients (20%) with regard to brain surgery. Global HRQoL was inversely correlated to DR (*r* = -0.35, *p* < 0.001). Physical functioning (*r* = -0.33, *p* < 0.001) and social functioning (*r* = -0.32, *p* < 0.001) demonstrated moderate correlation. As symptoms, fatigue (*r* = 0.32, *p* < 0.001), future uncertainty (*r* = 0.37, *p* < 0.001), motor dysfunction (*r* = 0.32, *p* < 0.001), depression (*r* = 0.37, *p* < 0.001) and satisfaction with medical care (*r* = -0.39, *p* < 0.001) showed strong correlations with DR. In multivariable linear regression, low satisfaction with medical care, unemployment, future uncertainty and BM stayed associated with DR.

**Conclusions:**

DR is frequent in patients with BT. Rather than treatment-related, low satisfaction with medical care and unemployment/financial uncertainty appear particularly linked to higher DR.

**Supplementary Information:**

The online version contains supplementary material available at 10.1007/s11060-026-05534-2.

## Introduction

Primary brain tumors and brain metastases are associated with significant morbidity and mortality. Despite treatment, malignant gliomas are still mostly not curable, with a median survival time of several years for low-grade gliomas and around 10–16 months for high-grade gliomas [1, 2]. Except for selected subgroups with targetable gene alterations, the overall survival of patients with brain metastases is generally measured also in months [3]. Besides this burden of an eventually fatal disease and often disabling neurological symptoms, patients also carry the weight of intensive treatments. Acute and late effects of chemoradiotherapy in malignant glioma and radiotherapy and differing systemic treatments in, usually heavily pre-treated, brain metastases patients may also negatively interfere with health-related quality of life (HRQoL) in the short and long run [4, 5]. Against this backdrop, patients can develop significant decision regret (DR). DR is defined as dissatisfaction or distress with a healthcare decision in the past [6, 7].

DR may be influenced by multiple factors, like treatment-related toxicities, disease progression, psychological distress, and involvement in decision-making [8–10]. In brain tumor patients, the concept of decision regret after treatment has not been analyzed so far, while there is increasing data in patients with other tumor types, like head and neck cancer [11]. High levels of DR may negatively affect emotional, social and physical well-being. Exemplary, in recent studies, DR was associated with anxiety and stress, and inversely correlated to HRQoL in head and neck cancer [12]. Further, elderly patients with different cancer types experiencing higher DR reported significantly higher concurrent symptom severity, lower functional scores, and lower treatment satisfaction [13]. With increasing treatment options, shared decision making (SDM) becomes more important and DR might help to identify gaps in the SDM process. DR might indicate that patient lacked adequate information or had limited time and generally experienced a mismatch between the patient’s preferred role in decision-making and their actual experience [14, 14]. By measuring regret, healthcare providers could identify which patients need more support to prevent long-term distress. Specifically, in patients with high-grade glioma or brain metastases in palliative treatment situations the impact of side effects of intensive chemoradiotherapy or whole brain radiotherapy (WBRT) in patients with multiple brain metastases could make these patients (and potentially their families) particularly prone to the feeling of DR after treatment, with relevant negative implications for their psychological well-being.

To address this aspect, this explorative bicentric study aimed to assess the prevalence of DR in patients with high- and low-grade glioma and brain metastases with consideration of different treatment components (i.e., radiotherapy, systemic treatment, and surgery). In addition, we aimed to reveal potential associations between DR and HRQoL, social variables, distress, anxiety, depression, and satisfaction with medical care.

## Methods

### Study design

This bicentric cross-sectional observational study was conducted at the Departments of Radiation Oncology of the University Medical Centers in Leipzig and Jena, Germany. Patients were eligible if (i) they had initiated radiotherapy for a brain tumor between 2010 and 2024, (ii) had completed at least one structured follow-up assessment, (iii) had provided informed written consent to study participation.

This study was performed in line with the principles of the Declaration of Helsinki. Approval was granted by the Ethics Committee of University of Leipzig (Date 4thApril 2024/ reference numbers: 372/23-ek and 077/23-ek) and the Ethics Committee of University of Jena (10.04.2025/ reference numbers: 2025-3746-Bef).

All procedures were performed in accordance with the Declaration of Helsinki, and the study was reported in line with STROBE guidelines for reporting observational research.

During the period December 2023 and July 2025, potentially eligible participants were approached by telephone and invited to participate in the study. After agreeing, participants received study information, a consent form, and paper-based questionnaires by post. Patient and treatment-related information were retrospectively extracted from electronic health records. Tumor grading was classified according to the 5th edition of the World Health Organization (WHO) Classification of Tumors of the Central Nervous System.

### Questionnaires

DR was assessed using the 5-item Decision Regret Scale (DRS) ranging from 1 (strongly agree) to 5 (strongly disagree [15]. If patients completed at least three of the five items of a DRS questionnaire (radiotherapy, concomitant systemic treatment or surgery, incl. biopsy), the mean value of the completed items was used for imputation for the missing values. Patients’ regret was evaluated separately for the last decision concerning radiotherapy, systemic treatment, and/or surgical resection with patients receiving separate DR questionnaires for each treatment modality. The original German wording of the DRS was: “Bitte denken Sie an die Therapieentscheidungen, eine Strahlentherapie zur Behandlung Ihrer Krebserkrankung durchzuführen.“ The English translation is: „Please consider your treatment decision regarding radiotherapy for treatment of your cancer.“ The same item stem was used for other treatment modalities, with the term “Strahlentherapie” replaced by the respective treatment (e.g., surgery or systemic therapy). The items were intended to capture regret related to the treatment decision itself, irrespective of whether patients ultimately underwent the respective treatment or decided against it. In accordance with the validated scoring algorithm of the DRS, DR was classified as absent (0 points), mild (1–25 points), and strong (> 25 points) [6]. An overall DRS score was calculated for patients who had completed at least two of the three DRS questionnaires. This overall score was defined as the mean score of those (two or three) DRS subscales that were completed by the patient. Health-related quality of life (HRQoL) was measured with the European Organization for Research and Treatment of Cancer (EORTC) QLQ-C30 questionnaire [16]. To capture brain tumor-specific patient-reported symptom burden, the EORTC BN20 questionnaire was additionally administered [17]. Distress was evaluated with the German version of the National Comprehensive Cancer Network (NCCN) distress thermometer, ranging from 0 (no distress) to 10 (extreme distress) [18]. Anxiety was measured using the 7-item General Anxiety Disorder scale (GAD-7) [19] with imputation applied, if at least four items were completed. Depression was assessed using the Patient Health Questionnaire 9-item scale (PHQ-9) [20]. If five or more items were answered, the mean of the completed items was used for imputation. Satisfaction with medical care was quantified using the question “Were you satisfied with medical care?” with a five-point Likert scale ranging from 1 (strongly disagree) to 5 (strongly agree). Proxy completion was allowed for all provided questionnaires and impaired patients were allowed assistance.

### Statistical analysis

All statistical analyses were performed using IBM SPSS Statistics 29 (IBM Corp., Armonk, NY, USA). Figures were created using Microsoft^®^ PowerPoint for Mac Version 16.101.3 (Microsoft Corporation, Redmond, WA, USA). Descriptive statistics were used to summarize the study cohort, including measures of central tendency (mean, median) and dispersion (standard deviation [SD], interquartile range [IQR]) for continuous variables.

Associations between DR and covariates were examined in univariate analyses using Spearman’s *ρ*, Pearson’s *r*., or one-way analysis of variance (ANOVA), depending on the measurement scale.

Cronbach’s *α* was used to verify the internal consistency of the 5-item DRS. For multivariable analyses, linear regression models were calculated using listwise deletion of missing data. Variables significantly associated with DR in the univariate analysis, or showing an at least moderate correlation (defined by *r* ≥ 0.35), were simultaneously entered into a linear regression model with DR as the dependent variable. For the purpose of this analysis, employment status was recorded into a binary variable with two categories (employed versus not employed). Statistical significance was defined as *p* < 0.05. Due to the exploratory character of the study, no correction for multiple testing was performed.

## Results

### Study cohort

**Fig. 1:**
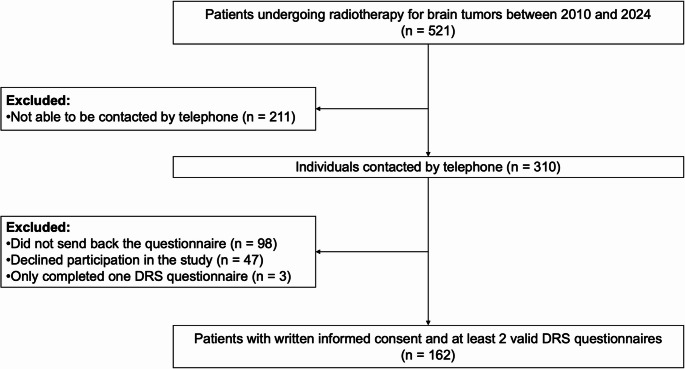
Study cohort flow diagram DRS, Decision Regret Scale

Out of 521 eligible patients who were contacted, total of 310 were reached by telephone. Among these, 263 agreed to participate, 47 declined, and 98 did not return the questionnaire. A total of 165 (165/310; 53%) patients returned the completed questionnaire. After excluding three participants who provided only one DRS form, 162 patients remained in the study (Fig. 1).

Patient and treatment characteristics of the study cohort are summarized in Table 1. Participants had a median age of 58 years (IQR, 46–67) at the start of radiotherapy. The gender distribution was balanced with 79 men (49%) and 83 women (51%). The majority of patients had primary brain tumors (*n* = 94, 58%), of which 70/162 (43%) were classified as WHO grade ≥ 3. Among the 68 patients (42%) with brain metastases secondary to tumors outside of the brain, the most common primary sites were the lung (*n* = 30, 19%) and the skin (*n* = 16, 10%). Most patients demonstrated a favorable performance status (Eastern Cooperative Oncology Group (ECOG) ≤ 1; *n* = 142, 88%). All participants underwent radiotherapy, of which the majority received conformal techniques (Three-Dimensional Conformal Radiation Therapy (3D-CRT), Intensity-Modulated Radiation Therapy (IMRT), Volumetric Modulated Arc Therapy (VMAT)) (*n* = 99, 61%) for their last course of radiation. Systemic treatment during/after radiotherapy was administered to 95 patients (59%), and 111 patients had a surgical resection of their brain tumor (69%). The median interval between the last radiotherapy fraction and study participation was 22 months (IQR, 8–47). HRQoL, assessed via the EORTC QLQ-C30 “general health/QoL” scale, had a mean score of 57.6 (SD 22.9).

**Table 1:** Patient and treatment characteristics (*n* = 162)

			Median (IQR)^a^
Age at the start of first radiotherapy course [years]			58 (46–67)
Radiotherapy treatment fractions			17 (3–30)
Total radiation dose [Gy]			40.9 (20.8–60)
Time between last fraction of radiotherapy and study participation [months]			22 (8–47)
		**n**	**%**
Gender			
	Male	79	49
	Female	83	51
Employment status			
	Employed	60	37
	Not employed	30	19
	Retired	58	36
	Unknown	14	9
Performance status			
	ECOG^b^ 0	71	44
	ECOG 1	71	44
	ECOG 2	13	8
	ECOG 3	5	3
	Unknown	2	1
Primary tumor localization			
	Brain	94	58
	Lung	30	19
	Skin	16	10
	Other	22	14
Grade/Brain metastasis			
	WHO Grade 1	3	2
	WHO Grade 2	21	13
	WHO Grade 3	33	20
	WHO Grade 4	37	23
	Brain Metastasis	68	42
Target volume of brain irradiation			
	Frontal lobe	37	23
	Temporal lobe	31	19
	Parietal lobe	9	6
	Occipital lobe	5	3
	Deep structures	12	7
	More than one target volume within the brain	39	24
	Whole brain	11	7
	Other	18	11
Type of radiotherapy			
	3D-CRT^c^/ IMRT^d^/ VMAT^e^	99	61
	Stereotactic radiotherapy	52	32
	Whole brain radiation therapy	11	7
Systemic treatment during or after radiotherapy of a brain tumor			
	Systemic treatment	95	59
	No systemic treatment	67	41
Surgical resection of a brain tumor			
	Complete resection	69	43
	Partial resection	42	26
	Biopsy	5	3
	No surgical resection	46	28

Abbreviations: ^a^IQR, interquartile range; ^b^ECOG, Eastern Cooperative Oncology Group; ^c^3D-CRT, Three-Dimensional Conformal Radiation Therapy; ^d^IMRT, Intensity-Modulated Radiation Therapy; ^e^VMAT, Volumetric Modulated Arc Therapy

Numbers may not add up to 100% due to rounding

### Prevalence of DR

**Fig. 2:**
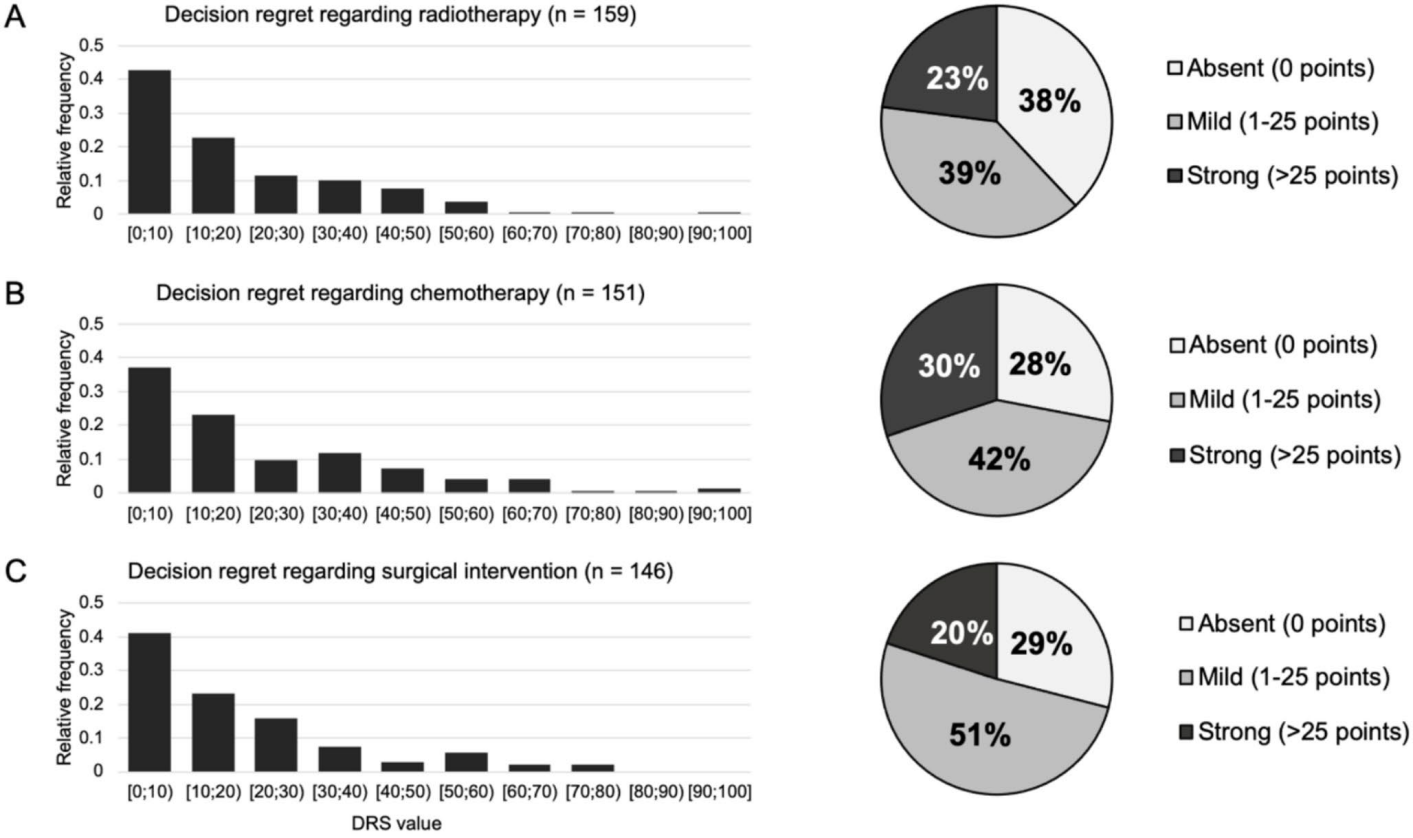
Frequency of decision regret regarding radiotherapy (**A**), systemic treatment (**B**), and surgical intervention (**C**) Histogram showing relative frequencies regarding the level of decision regret, as assessed using the 5-item Decision Regret Scale (DRS). Pie chart showing the frequency of absent (0 points), mild (1–25 points), and strong (> 25 points) decision regret. Square brackets indicate that the value is included, while parentheses mean that the value is excluded. For example, [0,10) represents the range from 0 to 9, including 0 but excluding 10

For this study, it was assumed that all patients completed the DR questionnaire, regardless of whether they received the corresponding therapy for their brain tumor. Of 162 patients receiving radiotherapy, a total of 159 (98%) completed the DRS regarding radiotherapy. Among them, the mean DR was 15.5 points (SD 17.5). In detail, 60 patients (38%) reported no DR (0 points), 62 (39%) indicated mild regret (1–25 points), and 37 (23%) expressed pronounced DR (> 25 points) (Fig. 2). Of the 159 patients who completed the DRS regarding radiotherapy, there were 158 patients with complete data and one patient with imputation due to 1–2 missing items.

The DRS for systemic treatment was completed by 151 patients, yielding a mean DRS score of 19.2 points (SD 20.8). 42 patients (28%) showed no DR, 64 (42%) noted mild regret, and 45 (30%) reported high DR regarding systemic treatment. The 151 patients were composed of 147 patients with complete item sets and four patients with 1–2 missing items.

Among the 146 respondents who completed the DRS, related to brain surgery the mean DR score reached 16.0 points (SD 17.5). Results showed that 42 patients (29%) experienced no DR, whereas 75 patients (51%) reported mild and 29 patients (20%) strong DR. A total of 143 patients provided complete data, and three patients had 1–2 missings. Due to the low proportion of patients with missing data (between 1 and 4 patients), it seems not necessary to calculate separate analyses for patients with missing data.

In comparison of the three mean scores of DR, only the difference between DR regarding radiotherapy and systemic therapy was statistically significant (*p* = 0.003), while the other two pairs, surgery vs. systemic therapy (*p* = 0.198) and surgery vs. radiotherapy (*p* = 0.687), showed no significant differences.

The overall DRS score, based on the mean of the three single scale means (*n* = 162), noted a mean DR of 17.3 (SD 16.7). Distribution was: 32 patients (20%) with no overall DR, 85 (53%) with mild, and 45 (28%) with a high overall DR.

The reliability coefficients (Cronbach’s *α*) of the DRS items were: *α* = 0.70 (radiotherapy), *α* = 0.78 (systemic therapy), and *α* = 0.70 (surgery). The combined DRS with 15 items yielded an *α* of 0.85.

Strong correlations were observed between the DRS scores across treatment modalities, with the highest correlation between radiotherapy and systemic treatment (*r* = 0.67, *p* < 0.001), followed by that between surgical intervention and systemic treatment (*r* = 0.57, *p* < 0.001) and the lowest correlation between radiotherapy and surgical intervention (*r* = 0.51, *p* < 0.001).

The figures reported above refer to all patients, irrespective of whether they received the special treatment or not. All patients received radiotherapy, but only 95 patients received systemic therapy, and 111 patients had a surgical resection. When comparing the 95 patients with systemic therapy with those not receiving systemic therapy, the mean DRS scores (regarding systemic therapy) were 18.3 (SD 19.0) and 20.6 (SD 23.4), respectively. The comparison between the 65 patients receiving surgery with those patients not receiving that treatment yields the following mean scores: 15.5 (SD 17.2) vs. 17.5 (SD 18.5). In both cases, systemic therapy and surgery, the effect size of the group comparison was *d* = 0.11, and the differences were not statistically significant (*p* = 0.520 and *p* = 0.541, respectively).

In all, 65 of the patients received all of the three kinds of treatment and completed all the corresponding questionnaires. The mean scores of these complete receivers were as follows: DRS radiation therapy: (13.5 (SD 13.8); systemic therapy: 16.3 (SD 16.5), and DRS-surgery: 13.4 (SD 16.7).

Regarding non-participants, data from 47 patients was available. Suppl. Table 2 presents characteristics of this group. When compared with the study group, the non-participants were 9 years older (t-test; *p* < 0.001). Further, the ECOG performance status was significantly less favorable in the group of non-participants (Chi²-test, *p* < 0.001), and among those patients who had no brain metastases, the mean WHO grade was also less favorable in the non-participants’ group (t-test, *p* = 0.015). There were no significant gender differences (Chi²-test, *p* = 0.755), no differences in tumor localization (Chi²-test, *p* = 0.503), and no significantly different proportions of patients with brain metastases (Chi²-test, *p* = 0.173). Most frequent reason for declining to participate was excessive physical strain.

#### Association of DR with patient-reported outcomes and clinical variables

Analysis of patient-reported outcomes indicated correlations between DR (overall score, i.e. mean of the three single scale means) and HRQoL (*r* = -0.35) (Table 2). Among the other functioning EORTC QLQ-C30 scales, only physical functioning (*r* = -0.33) and social functioning (*r* = -0.32) demonstrated moderate correlation with Pearson’s *r* values above 0.3. Within the symptom scales, fatigue (*r* = 0.32) exhibited the strongest correlation with DR. Of the BN20 scales, future uncertainty (*r* = 0.37) and motor dysfunction (*r* = 0.32) showed the highest associations with DR.

Depression (*r* = 0.37) was also correlated above the 0.3 threshold, whereas anxiety (*r* = 0.25) and distress.

(*r* = 0.29) demonstrated weaker correlations. Among all patient-reported outcomes, satisfaction with medical care (*r* = -0.39) showed the strongest overall correlation with DR.

**Table 2:** Correlation between general DR and other patient-reported outcome measures

Variable	*R*	*p*	*n*
**EORTC QLQ-C30**			
Global health/QoL	**-0.35**	**< 0.001**	159
*Functioning scales*			
Physical	**-0.33**	**< 0.001**	162
Role	-0.22	< 0.005	162
Emotional	-0.28	< 0.001	161
Cognitive	-0.15	0.059	161
Social	**-0.32**	**< 0.001**	161
*Symptom scales*			
Fatigue	**0.32**	**< 0.001**	161
Nausea/Vomiting	0.28	< 0.001	162
Pain	0.20	0.010	162
Dyspnea	0.17	0.028	161
Insomnia	0.25	0.001	162
Appetite loss	0.24	0.002	162
Constipation	0.15	0.052	162
Diarrhea	0.05	0.503	162
Financial difficulties	0.15	0.051	160
**EORTC QLQ-BN20**			
Future uncertainty	**0.37**	**< 0.001**	161
Visual disorder	0.27	< 0.001	161
Motor dysfunction	**0.32**	**< 0.001**	161
Communication deficit	0.14	0.084	161
Headaches	0.22	< 0.005	160
Seizures	0.17	0.035	160
Drowsiness	0.16	0.041	160
Hair loss	0.29	< 0.001	161
Itchy skin	0.12	0.143	160
Weakness of legs	0.22	< 0.005	161
Bladder control	0.28	< 0.001	161
**Further variables**			
Depression (PHQ-9)	**0.37**	**< 0.001**	162
Anxiety (GAD-7)	0.25	0.001	162
Distress	0.29	< 0.001	157
Satisfaction with medical care	**-0.39**	**< 0.001**	162

Correlations with *r* ≥ 0.3 are shown in bold

No significant associations were found between DR and age (*r* = 0.15, *p* = 0.066) or gender (Table 3; *p* = 0.103). Employed patients reported significantly lower levels of DR than patients who were not employed (*p* = 0.012).

The number of treatment fractions (*r* = -0.12, *p* = 0.131), the total radiation dose (*r* = -0.12, *p* = 0.134), and the time between last fraction of radiotherapy and study participation (*r* = -0.01, *p* = 0.886) had no significant impact on DR. One-way ANOVAs did not reveal significant associations between DR and categorical clinical variables such as performance status, tumor grading/brain metastasis, intracranial tumor location, type of radiotherapy, as well as administration of systemic treatment or surgical resection of the brain tumor (Table 3). However, patients with brain metastases reported significantly higher levels of DR than patients with primary brain tumors (*p* = 0.025).

**Table 3:** Association of overall DR and categorical independent variables per one-way ANOVA (*n* = 162) Overall DR was assessed with the 5-item Decision Regret Scale (DRS) as mean of the three treatment related scale means

		*n*	Mean	SD^a^	*p*
Gender					
	Male	79	15.1	14.8	
	Female	83	19.4	18.1	0.103
Employment status					
	Employed	60	12.6	12.0	
	Not employed	30	19.4	15.1	
	Retired	58	20.9	18.4	0.012
Performance Status					
	ECOG^b^ 0	71	15.0	15.6	
	ECOG 1	71	19.1	16.9	
	ECOG 2	13	19.1	19.3	
	ECOG 3	5	19.3	22.2	0.505
Grade/Brain metastasis					
	WHO grade 1	3	16.7	7.6	
	WHO grade 2	21	15.5	14.8	
	WHO grade 3	33	16.0	14.2	
	WHO grade 4	37	13.3	13.4	
	Metastasis	68	20.8	19.7	0.238
Primary tumor localization					
	Brain	94	14.8	13.7	
	Other	68	20.8	19.7	0.025
Intracranial tumor location					
	Frontal lobe	37	17.6	16.8	
	Temporal lobe	31	17.6	14.3	
	Parietal lobe	9	10.5	16.4	
	Occipital lobe	5	21.3	22.0	
	Deep structures	12	22.4	17.3	
	More than one target volume within the brain	39	13.8	15.6	0.541
	Whole brain	11	19.8	17.5	
	Other	18	21.4	20.4	
Type of radiotherapy					
	3D-CRT^c^/ IMRT^d^/ VMAT^e^	99	16.7	15.5	
	Stereotactic radiotherapy	52	18.1	18.8	
	Whole brain radiation therapy	11	19.8	17.5	0.783
Systemic treatment of brain tumor/brain metastasis					
	Systemic treatment	95	15.7	14.2	
	No systemic treatment	67	19.6	19.6	0.143
Surgical resection of a brain tumor					
	Complete resection	69	15.3	13.7	
	Partial resection	42	17.7	17.2	
	Biopsy	5	11.7	16.7	
	No surgical resection of a brain tumor	46	20.6	19.9	0.345

Abbreviations: ^a^SD, standard deviation; ^b^ECOG, Eastern Cooperative Oncology Group; ^c^3D-CRT, Three-Dimensional Conformal Radiation Therapy; ^d^IMRT, Intensity-Modulated Radiation Therapy; ^e^VMAT, Volumetric Modulated Arc Therapy

The combined impact of multiple factors on DR was calculated in terms of a multivariate regression analysis.

Criteria for the inclusion in the model were as follows: Among the categorical variables (Table 3), those with a significant association with DR were included: employment status and primary tumor localization (brain or others). Among the continuous variables (Table 2), the variables with the highest correlation with DR (correlations of 0.35 and above) were included. There were multiple other variables with a strongly significant association with DR (11 variables with *p* < 0.001), however, all variables listed in Table 2 are more or less interrelated, and in order to reduce the effect of collinearity we restricted the analysis to these four variables. The correlations among the four continuous variables entered in the regression model were between *r* = 0.18 (global health/QoL and satisfaction with medical care) and *r* = -0.68 (global health/QoL and PHQ-9 depression).

Finally, global Health/QoL, future uncertainty, depression, satisfaction with medical care, employment status, and brain metastases were simultaneously included in a linear regression analysis (*n* = 144). Supplementary Table 1 presents the results. The total model predicted significant DR (F(6,138) = 9.585, *p* < 0.001). Higher levels of DR were observed among patients reporting dissatisfaction with medical care (*β* = − 0.332), future uncertainty *(β* = 0.232*)*, unemployed patients (*β* = 0.167), and those with brain metastasis (*β* = 0.153*).*

With low VIF factors (Max. = 2.3) and high tolerance values (Min. = 0.433) there was low indication of collinearity of the included variables.

## Discussion

To the best of our knowledge, this is the first systematic examination of decision regret (DR) in patients with primary brain tumors or brain metastases. Overall, the impression of at least mild DR was prevalent. Only 20% of patients reported no overall DR, while 53% reported mild DR and 28% reported severe DR. To better contextualize these results, it is necessary to make a rough comparison with patients with other tumor types. The frequency of DR in this cohort of brain tumor patients is consistent with the results of other studies conducted by our group. In a cross-sectional analysis of patients with various tumor types, the prevalence of strong DR was 18% [15]. Among patients with head and neck cancers, more patients (39%) reported strong DR after treatment.

Within the here examined cohort the median time from radiotherapy was 22 months, predominant ECOG was 0–1 and median patient age was 58 years. Compared to patients that did not participate median age and ECOG were lower. These cohort features imply the selection a favorable subset of patients for reporting of DR. Survival in the majority of patients with malignant glioma and brain metastasis is often much poorer [2, 21]. It needs to be stated that for short-term survivors with malignant brain tumors these results and conclusions cannot be easily transferred. Further, the patient cohort including different tumor entities was deliberately chosen to be heterogenous in order to mirror DR in a broader neuro-oncologic patient population. However, presented results must be seen as hypothesis generating and require further tumor-specific validation.

In our analysis, the extent of DR varied between decisions towards certain treatments: strong DR was reported by 23% of patients for the decision for/against radiotherapy, 30% for the decision for/against chemotherapy, and 20% for the decision for/against surgery. It is known that radiotherapy can cause symptoms such as fatigue and neurocognitive decline, which can have a negative impact on HRQoL [4, 22]. However, focal radiotherapy is generally well tolerated in large clinical trials without severely impacting HRQoL or neurocognition [23, 24]. Of the patients in our analysis with BM, only 11 received whole-brain radiotherapy (WBRT) as their last treatment, which is the potentially most burdensome type of cerebral radiotherapy. Compared with focal radiotherapy, WBRT has been associated with greater cognitive deterioration and poorer HRQoL in patients with BM in large clinical trials [25]. In our analysis, DR was not higher in patients after WBRT than in those treated with focal radiotherapy. However, the very limited sample size means that no firm conclusions can be drawn. Further, the time interval of 22 months from last radiotherapy is too short to cover consequences of potential long-term side effects of radiotherapy.

Regarding systemic treatment, it is also difficult to draw conclusions about specific treatments in our mixed patient cohort. For example, treatment with lomustine, procarbazine and vincristine (PCV) is known to be associated with significant, potentially persistent side effects and greater toxicity than temozolomide treatment [26, 27]. This may have theoretically contributed to a higher degree of DR with respect to decision regarding systemic treatment in our cohort. However, DR with regard to different treatments was highly correlated, and perceptions of DR were mostly unrelated to the type of treatment. The context of HRQoL appeared to be a much more relevant factor in the perception of DR. Generally, an inverse correlation was observed between DR and HRQoL. Among the EORTC QLQ-C30 subscales, physical and social functioning showed a moderate correlation with DR, as did the fatigue symptom scale. Among the BN20 scales, future uncertainty and motor dysfunction exhibited the strongest associations with DR. Among the other scales, depression also showed a correlation above the 0.3 threshold, whereas anxiety and distress demonstrated weaker correlations. These findings resemble those observed in other tumor entities, such as head and neck or prostate cancer, where strong associations between HRQoL and DR were evident [12, 15, 28, 29]. It appears likely that reduced HRQoL can aggravate DR, also in brain tumor patients. Reduced physical and social functioning, as well as fatigue in particular, may lead to a negative perception of past treatment decisions and more intense DR. Physical and mental fatigue are particularly disabling for patients’ lives and might therefore also impact DR [5].No significant associations were found between DR and continuous clinical variables, including age and the time elapsed between the last radiotherapy session and participation in the study. Furthermore, univariate analyses revealed no significant associations between DR and variables such as gender, performance status and primary tumor localization. The lack of impact of age and ECOG status is notable. While these factors are highly relevant to caregivers for prognostic assessment, treatment decisions and estimation of well-being, they may not influence patients’ highly individual judgement of DR.

With regard to tumor types, patients with brain metastases reported higher DR than patients with primary brain tumors. In addition to the psychosocial burden from this often rapidly fatal disease, treatment-related loss of HRQoL might be particularly relevant in these patients [30].

A confounder for potentially more DR among patients with brain metastasis is related to potentially more available treatment options. The “paradox of choice” i.e. the availability of more options with more perceived responsibility for the choice and potentially more regret, could be applicable [31]. So far, evidence for the applicability of this phenomenon concerning DR after treatment decisions in oncology is lacking.

Overall, the reasons for higher DR in patients with BM require validation in larger, more specific studies.

Regarding socioeconomic variables, patients who were unemployed at the time of the study reported higher levels of DR than those in employment, in both univariable and multivariable analyses. Approximately 50% of patients return to work following treatment for low-grade glioma, compared to around 20% following treatment for high-grade glioma [32, 33]. Loss of employment and the subsequent financial uncertainty are known to significantly contribute to distress and poorer HRQoL in glioma patients. Therefore, an association with DR seems reasonable [34]. To tackle the issue of financial uncertainty, comprehensive counselling on rehabilitation options, social security disability benefits and other financial support would be beneficial. There is very limited data from the US concerning social security disability benefits for patients with low-grade glioma, and much more research is needed in this highly relevant field [35].

The presence of depression did not remain associated with a significant effect on DR in multivariate linear regression analysis. This appears somewhat surprising, as depression has a high point prevalence of 20–40% in glioma patients and is highly negatively associated with HRQoL and even prognosis in glioma patients [36]. Furthermore, the multivariate analysis revealed that reduced satisfaction with medical care was significantly linked to higher DR. This finding is particularly relevant given that satisfaction with medical care is a factor that can be easily modified from the caregivers’ perspective. Studies in the palliative care of high-grade glioma patients have shown that satisfaction with the information provided and effective symptom treatment are highly associated with satisfaction with medical care in general [37]. Improving symptomatic treatment and information transfer to patients and families by the help of information material, digital tools and enhanced SDM could be powerful interventions to reduce DR [38].

Our study cannot establish causal links, but it appears plausible that factors like reduced HRQoL, depression, satisfaction with medical care and socioeconomic factors interact with DR and vice versa. Decision regret is often accompanied by negative emotions such as guilt or disappointment, which may lead to anxiety or depression [12, 39]. In a prospective cohort study with surgically treated head-and-neck cancer patients, moderate to severe decision regret was associated with baseline frailty, higher depression scores, reduced instrumental activities of daily living, and lower psychological well-being [40]. More and longitudinal studies are needed to better understand the phenomenon of DR in brain tumor patients and measures to counteract.

We think, that the separate measurement of DR is important beyond the common evaluation of HRQoL. Patients with high-level of DR should be specifically approached in order to identify individual factors that attributed to DR in this patient. Discussing contributing factors with the patients might mitigate DR and improve individual perceptions of past treatment decisions. This might be particularly relevant in patients with brain tumors with neurologic deficits or palliative situations in order to alleviate associated distress.

Exemplary, if a patient regrets the decision regarding a specific type of treatment it might help to retrospectively reflect the alternatives that were available at that time of the decision in order to put the received treatment into the context of potential other options with their disadvantages. Further, a systematic evaluation of DR among patients might help to detect shortcomings in the decision-making process regarding specific treatments. To facilitate optimal, patient-centered decisions, it is thought that patients should have an adequate understanding of their treatment options. Providing information about different anti-cancer treatments in a comprehensive way appears crucial [41, 42]. Further, it is thought, that assisting patients in adopting individually preferred more active or passive modes in SDM might lead to low levels of DR [43]. Ultimately, perception of emotional support, care and appreciation of personal values from health providers appears a valid strategy do reduce DR (discussed in [14]).This study examined decision regret not only in general terms, but specifically in relation to different forms of therapy. Our study also examined the extent to which decision regret is present in patients who have decided against therapy. In our view, it is relevant to consider not only the therapies that were undergone, but also those that were consciously not taken up in terms of decision regret.

When comparing patients who received a certain therapy with those who did not, regret about the decision was slightly higher on average among those who did not receive the therapy, although the differences between the groups were small and insignificant. It is therefore not possible to make generalizations.

However, this procedure poses methodological difficulties in that different measures of decision regret are based on different subsamples. Limiting the study to those patients who underwent all three forms of therapy and who completed the DRS for all of these forms of therapy allows for a better comparison between the forms of therapy, but has the disadvantage that the sample size is reduced to 38% of the original sample size, and that statements about patients who underwent fewer than these three forms of therapy are therefore not possible.

The difficulties in determining therapy-specific decision regret shown here, as well as the high correlations between these components of decision regret (r between 0.51 and 0.67), suggest that a global assessment of decision regret, as is commonly done, has advantages over specific questioning, not only because of its economy (only 5 questions), but also because of its clarity of content.

Despite the relevance of our findings, the study has several limitations. Due to the cross-sectional design, the longitudinal assessments necessary for the exact causal attribution of DR to specific treatment types or occurrences of tumor progressions are unavailable. As mentioned a selection of long-term survivors among patients with high-grade glioma and brain metastasis is present. Patients who died within a short timeframe, who may have had a higher DR, are underrepresented in our analysis. Most likely, higher DR may have been more prevalent among patients who did not return the questionnaires. To obtain a more granular, tumor-specific and also intercultural perspective, larger international datasets are necessary, which are being planned by our group. This is particularly relevant for patients with brain metastases. These patients constituted only a very limited number in our analysis. Furthermore, although we examined numerous clinical and psychological variables, other potential contributors to DR, such as health literacy, social support and fear of cancer recurrence, which have been identified as significant factors in prior research, were not included in our study and should be considered in future analyses [12, 15, 44, 45].

Another -statistical- problem is that (as in other studies on DRS) the distribution deviates significantly from a normal distribution, and therefore the requirements for some of the statistical methods are not exactly met. Despite these limitations, our findings provide the first systematic data on DR in brain tumor patients, which should be augmented by further studies. Our data suggest that patients’ DR is associated with physical and socioeconomic well-being, particularly being driven by modifiable factors such as low satisfaction with medical care, future uncertainty and unemployment.

## Supplementary Information

Below is the link to the electronic supplementary material.

Supplementary Material 1

## Data Availability

The datasets generated during and/or analysed during the current study are available from the corresponding author on reasonable request.
