# Supplementary material for: Regret concerning treatment decisions in patients with primary or secondary brain tumors – a cross-sectional exploratory bicentric analysis
Source: J Neurooncol. 2026 Mar 25;177(2):74. doi: 10.1007/s11060-026-05534-2 (PMC13018084; doi:10.1007/s11060-026-05534-2)
Supplement: Supplementary file 1 — Supplementary Material 1 [file 11060_2026_5534_MOESM1_ESM.docx]

**Suppl. Table 1 Predictors of DR per multiple linear regression analysis.** Complete-case analysis
(n = 145) with DR as the dependent variable. Confidence intervals are reported for the unstandardized regression coefficients (reference value = 0).

| **Variable** | ***β*** | **B** | **Lower 95% CI^a^** | **Upper 95% CI** | ***p*** |
| --- | --- | --- | --- | --- | --- |
| Global health/QoL^b^ | 0.017 | 0.012 | -0.139 | 0.163 | .873 |
| Future uncertainty | 0.232 | 0.127 | 0.018 | 0.237 | **.023** |
| Depression | 0.088 | 0.290 | -0.388 | 0.969 | .399 |
| Satisfaction with medical care | -0.332 | -6.414 | -9.219 | -3.609 | **<.001** |
| Unemployment | 0.167 | 5.344 | 0.465 | 10.223 | **.032** |
| Brain metastasis | 0.153 | 4.880 | 0.095 | 9.665 | **.046** |
| Abbreviations: **^a^**CI, confidence interval; ^b^QoL, quality of life. | | | | | |

**Suppl. Table 2 Non-Participant characteristics (n=47)**

|  |  | |  | Median (IQR)^a^ | |
| --- | --- | --- | --- | --- | --- |
| Age at refusal [years] |  | |  | 67 (56-73) | |
|  |  | |  |  | |
|  | |  | **n** | | **%** |
| Gender | |  |  | |  |
|  | | Male | 24 | | 51 |
|  | | Female | 23 | | 49 |
| Performance status | |  |  | |  |
|  | | ECOG^b^ 0 | 13 | | 28 |
|  | | ECOG 1 | 7 | | 15 |
|  | | ECOG 2 | 7 | | 15 |
|  | | ECOG 3 | 17 | | 36 |
|  | | ECOG 4 | 3 | | 6 |
| Primary tumor localization | |  |  | |  |
|  | | Brain | 22 | | 47 |
|  | | Lung | 13 | | 28 |
|  | | Skin | 4 | | 9 |
|  | | Other | 8 | | 17 |
| Grade/Brain metastasis | |  |  | |  |
|  | | WHO Grade 2 | 2 | | 4 |
|  | | WHO Grade 3 | 5 | | 11 |
|  | | WHO Grade 4 | 15 | | 32 |
|  | | Brain Metastasis | 25 | | 53 |
| Main reason for non-participation | |  |  | |  |
|  | | Excessive physical strain | 17 | | 36 |
|  | | Excessive mental strain | 3 | | 6 |
|  | | No interest | 6 | | 13 |
|  | | Logistical reasons | 10 | | 21 |
|  | | Other | 11 | | 23 |
| Abbreviations: ^a^IQR, interquartile range; ^b^ECOG, Eastern Cooperative Oncology Group  Numbers may not add up to 100 % due to rounding. | | | | | |
